# Gypsy endogenous retrovirus maintains potential infectivity in several species of Drosophilids

**DOI:** 10.1186/1471-2148-8-302

**Published:** 2008-10-31

**Authors:** Jose V Llorens, Jonathan B Clark, Isabel Martínez-Garay, Sirena Soriano, Rosa de Frutos, María J Martínez-Sebastián

**Affiliations:** 1 Departament de Genètica, Universitat de València, 46100-Burjassot, Valencia, Spain; 2 Department of Zoology, Weber State University, Ogden, 84408-2505 Utah, USA

## Abstract

**Background:**

Sequences homologous to the *gypsy *retroelement from *Drosophila melanogaster *are widely distributed among drosophilids. The structure of *gypsy *includes an open reading frame resembling the retroviral gene *env*, which is responsible for the infectious properties of retroviruses.

**Results:**

In this study we report molecular and phylogeny analysis of the complete *env *gene from ten species of the *obscura *group of the genus *Drosophila *and one species from the genus *Scaptomyza*.

**Conclusion:**

The results indicate that in most cases *env *sequences could produce a functional Env protein and therefore maintain the infectious capability of *gypsy *in these species.

## Background

*Gypsy *(*gypsyDm*) is an endogenous retrovirus of *D. melanogaster*. Its structure is largely similar to the vertebrate retroviruses and it possesses infective properties [1-4]. Sequences homologous to *gypsyDm *are widely distributed among *Drosophila *species, found in both the subgenus *Sophophora *as well as in the subgenus *Drosophila *[5-9]. In addition, they have been detected in some species of the genus *Scaptomyza *[8,9]. The phylogenetic relationships among *gypsy *sequences from *Drosophila *species is not be always coincident with those of their hosts, which could be indicative of horizontal transfer during evolutionary history [6,7,9,10]. Horizontal transfer has been invoked to explain the evolutionary patterns of several families of transposable elements [11-15]. In some cases, such as the P element, the horizontal transfer events are strongly documented [16-18]. However, the underlying mechanisms to horizontal transfer remain elusive.

In the case of *gypsyDm*, the horizontal transfer could be associated with the infectious properties of that element. One way to evaluate the potential impact of the *gypsy *infectious ability on the evolutionary history of *gypsy *sequences from *Drosophila*, is to analyze the infective ability of *gypsy *homologous sequences from species other than *D. melanogaster*.

The infectious ability of *gypsyDm *is associated with the expression of a retroviral envelope-like protein encoded by the *env *gene [2]. Structural analysis of the *env *region of a given *gypsy *sequence is the first step in determining its potential infectious ability. In previous reports, two complete *gypsy *elements from *D. subosbcura *and *D. virilis *were sequenced [10,19]. Although, the *env *region is preserved in its full-length, in both species single indels result in potentially truncated Env proteins [10]. However, as has been proposed in [4], it is possible that both, defective and complete fully functional *gypsy *elements, coexist in the genome of these species. This study describes the existence of full-length *env *genes in the genomes of *D. subosbcura *and *D. virilis*, as well as in the genomes of the several species closely related to *D. melanogaster*:*D. simulans, D. erecta, D. orena, D. teissieri*, and *D. yakuba*. Interestingly, these *gyspy *sequences can potentially invade the *D. melanogaster *genome, and escape the control normally exerted by the *flamenco *gene, whose product represses mobility[4].

It is generally accepted that species of the *obscura *group can be classified into five subgroups: the *pseudobscura *and *affinis *subgroups, consisting of Neartic species, the *subobscura *and *obscura *subgroups, mainly consisting of Paleartic species, and the *microlabis *subgroup encompassing Afrotropical species [20-22]. *Gypsy *sequences homologous to *gypsyDm *have been detected in several species of the Neartic and Paleartic subgroups, appearing as a monophyletic group that is highly diverged from the *gypsyDm *prototypic element [7,8]. With the exception of *D. guanche*, the 5' region of the *env *gene in all species examined could potentially produce a functional N-terminal region of the Env protein. To confirm the existence of potentially infective *gypsy *lineages other than *gypsyDm, gypsyDs*, and *gypsyDv*, we report here an analysis of the complete *env *gene of ten species of the *obscura *group and one species from the genus *Scaptomyza*. Most of the species analyzed contain intact copies of the *env *region and the *env *splicing sites, and *in vitro *can be translated into a protein of the predicted size.

## Results and discussion

Sequences homologous to *gypsy *from *D. melanogaster *are widely distributed among *Drosophila *species. In previous studies, the complete *gypsy *elements from *D. subobscura *and *D. virilis *have been sequenced [10]. These studies were carried out to understand the evolutionary behaviour of *gypsy *and indicate that the genetic organization of *gypsy *in those species is conserved. Moreover, in *D. melanogaster *do the *gypsy *elements have a complete functional *env *gene [10]. In the case of *D. virilis *and *D. subobscura*, it has been shown that the genomes of these species contain at least one copy of *gypsy *putatively encoding a complete envelope protein [4].

The main aim of this study is to examine whether potentially functional copies of the *env *region of *gypsy *exist in other species. This was accomplished by searching genomes for the presence of full-length *env *coding sequences and assessing their functionality.

### The analysis of the *env *sequences shows their infectious capability

**Figure 1:**
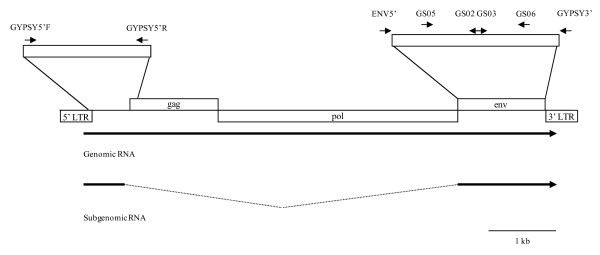
**Schematic diagram of the *gypsy *element.** The organization of the *gypsy *element and the genomic and subgenomic RNAs is shown along with the localization of the primers used in this study.

To analyze the infectious capability of the *gypsy *element of each analyzed species (table 1) we amplified the *env *region with specific primers designed from the intergenic sequence (figure 1). In all species the size of the amplified sequence was 1.5 kb, except for *D. algonquin *and *D. tolteca*, with sizes of 0.5 and 1.2 kb respectively. The PCR products of all species, were cloned in pCR^®^2.1-TOPO^® ^(Invitrogen) and sequenced with the specific primers of the vector, demonstrating that *D. algonquin *and *D. tolteca *had a deletion of 1.5 kb and 0.3 kb respectively. In the same way, *D. pseudoobscura *and *D. miranda *had deletions of 40 pb and 21 pb respectively that produced a stop codon and an Env truncated protein. *D. persimilis *had a single nucleotide change, also producing a stop codon. In contrast, *D. madeirensis*, *D. obscura*, *D. ambigua*, *D. bifasciata*, *D. affinis *and *S. elmoi *had different polymorphisms but none resulting in a truncated protein, so these *env *regions would produce potentially active proteins.

**Table 1:** List of species used in the analysis of *env *sequences.

Genus	Subgenus	Group	Subgroup	Species
*Drosophila*	*Sophophora*	*obscura*	*subobscura*	*D. madeirensis*
			*obscura*	*D. obscura*
				*D. ambigua*
				*D. bifasciata*
			*pseudoobscura*	*D. pseudoobscura*
				*D. persimilis*
				*D. miranda*
			*affinis*	*D. affinis*
				*D. algonquin*
				*D. tolteca*
*Scaptomyza*	*Parascaptomyza*			*S. elmoi*

With these data, we can conclude that *D. madeirensis*, *D. obscura*, *D. ambigua*, *D. bifasciata*, *D. affinis *and *S. elmoi *could produce a complete Env protein and therefore could maintain the infectious capability of *gypsy *in these species.

### The *env *sequences obtained in different species have the capacity to produce complete envelope proteins

The RTS system was used to scan for premature termination mutations and analyze the coding capacity of the *gypsy env *gene present in the genome of the different species studied. We used genomic DNA from each species to amplify the *env *sequence. We cloned the obtained sequences into pCR^®^2.1-TOPO (Invitrogen) and then we used these clones to produce the PCR products for *in vitro *protein expression (see material and methods).

**Figure 2:**
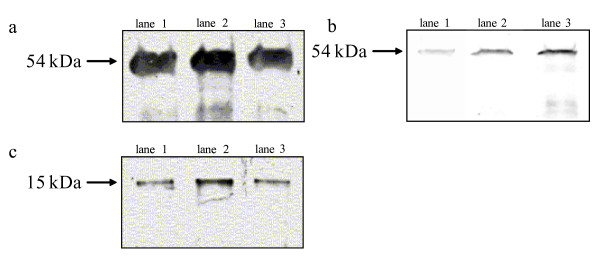
**In vitro protein sintesis using the RTS system.** a) and b) The proteins obtained, using complete sequences of *env *as templates had the sizes of normal Env protein (54 kDa). a) lane1, *D. madeirensis*; lane2, *D. obscura*; lane3, *D. ambigua*; and b) lane1, *D. bifasciata*; lane2, *D. affinis*; lane3, *S. elmoi*. c) The proteins obtained using disrupted sequences of *env *as templates were truncated (15 kDa). c) lane1, *D. pseudoobscura*; lane2, *D. persimilis*; lane3, *D. miranda*.

The major protein products obtained in this assay have molecular weights corresponding to the expected size for Env protein of *D. melanogaster*, about 54 kDa. These protein products were obtained in the same species where the *env *nucleotide sequence was complete, without disruptions. We obtained complete Env protein from *D. madeirensis*, *D. obscura*, *D. ambigua*, *D. bifasciata*, *D. affinis *and *S. elmoi*, while we obtained truncated proteins from the species where the sequence was disrupted, *D. pseudoobscura*, *D. persimilis *and *D. miranda *(figure 2).

In *D. melanogaster*, the splicing produces a start codon using AT from 5' site in the *gag *region and a G from the 3' site in the *env *region [2]. We have analyzed the sequences from the different species of this study and only *D. madeirensis, D. obscura, D. ambigua, D. bifasciata, D. affinis *and *S. elmoi*, have conserved the sequences that produce the start codon after the splicing.

**Figure 3:**
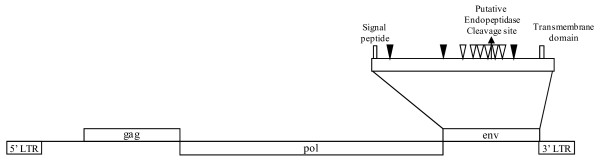
**Schematic structure of the functional *env *gene in the species analysed.** The location of the signal peptide of the precursor protein, the transmembrane domain, and the cellular endopeptidase cleavage site is indicated. The three asparagine (N) residues near the putative glycosylation sites are shown with filled arrowheads. The six cysteine (C) residues are denoted by open arrowheads.

These results show that these species contain at least one copy of *gypsy *element that can encode an envelope protein. Other data that support the existence of functional Env proteins include the presence of N-glycosylation sites, conserved cysteine residues and endopeptidase cleavage sites in all the species with a complete *env *sequence and the ATG codon (figure 3), as described in [2,4]. Moreover, those species have the sequence pattern (R-X(2)-R-X(5,6)- [GE]-X(5)- [LV]-X-G-X(2)-D-X(2)-D) for the detection *in silico *of endogenous retroviral envelope protein proposed in [23]. Only the signal peptide and the membrane-spanning domain sequence show differences on comparing to *D. melanogaster gypsy *sequence.

### Although sequences have the capability to produce complete envelope protein, their expression could be repressed in the species studied

To demonstrate the capability of the *gypsy *elements to produce an active envelope protein, we wanted to know if the *env *gene was expressed *in vivo *in the species with the complete *env *sequence.

In order to detect the production of the *env *mRNA we performed a RT-PCR analysis using the GS05 and GS06 primers with the mRNA extracted from ovarian chambers of the female flies. Since we also wanted to detect alternative splicing, necessary to produce ENV, we used the primers GYPSY5'F and GS06 of the *env *gene using the same mRNA as before (see materials and methods).

Amplification with the internal primers was successful in all species analysed as well as in the positive control (data not shown). However, alternative splicing was not detected (data not shown). These results suggest that the alternative splicing of *gypsy *in the *obscura *species group and *S. elmoi *would be repressed by a genomic gene as occurs in *D. melanogaster *with the *flamenco *gene [24]. Alternatively, the *gypsy *element may use an alternative start codon to express the *env *region or we simply could not detect the alternative splicing.

### The *gypsy *phylogeny is not totally consistent with species phylogeny

Since *gypsy *elements can potentially jump from one individual to another without the need of a vector, it has been proposed that horizontal transfers of *gypsy *elements is favoured by their ability to encoded full-length ENV proteins [4]. In this way we performed a phylogeny analysis of the *env gypsy *sequences studied, looking for evidences of horizontal transfer events.

**Figure 4:**
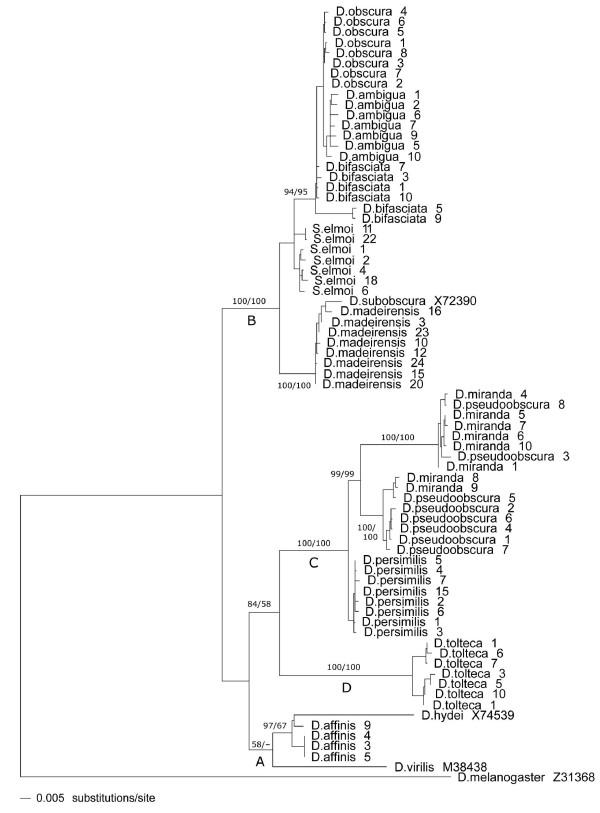
**Neighbor-Joining phylogeny of *gypsy *sequences from the *obscura *species group.** The four main clades, discussed in the text, are identified by letters. Numbers on branches represent bootstrap percentages from 1000 replicates as determined by Neighbor-Joining (value before slash) and parsimony (after slash). Values are shown only for the major clades. The scale bar denotes branch lengths in substitutions per site.

A Neighbor-joining phylogenetic tree of envelope sequences is shown in figure 4. The *obscura *sequences fall into four main clades that are generally consistent with species phylogeny. Clade A includes sequences from *D. affinis *of the *affinis *subgroup; clade B, *D. ambigua*, *D. bifasciata*, *D. madeirensis*, and *D. obscura *of the *obscura *subgroup; clade C, *D. miranda*, *D. persimilis*, and *D. pseudoobscura *of the *pseudoobscura *subgroup; and clade D, *D. tolteca *of the *affinis *subgroup. The *D. virilis *sequence is responsible for the relatively low support for clade A; removing the *D. virilis *sequence from the analysis increases the bootstrap values to 98/77 for a grouping of the *D. hydei *sequence with those from *D. affinis*, with the rest of the topology unchanged. With the exception of the placement of sequences from *S. elmoi*, the topology of this tree is identical to that obtained by parsimony analysis, which yielded a shortest tree of 1477 steps (Consistency Index = 0.8; Retention Index = 0.9). The distribution of sequence from *D. affinis *and *D. tolteca *into two distinct clades is an unexpected result that is consistently seen in all analyses. Constraining the analysis so that the sequences from D. tolteca and D. affinis are monophyletic adds an additional nine steps to the parsimony tree.

The potential effect of recombination on the phylogeny was assessed by using the GARD algorithm, which uses maximum likelihood models to detect recombination events. The BIC model found no evidence of recombination, while the AIC model identifies a potential breakpoint at position 1288 in the 1392 character alignment. The potential effect of this event on the phylogeny was also examined using a sliding-window approach. Separate phylogenetic analyses were constructed from four 400-bp regions of the envelop alignments (positions 1–399; 400–799; 800–1199; and 1200–1493), and on a fifth dataset that excluded the region beyond the breakpoint. The latter analysis resulted in an identical tree to that shown in figure 4, as did two of the four sliding window analyses. In the other two sliding-window trees, the *D. virilis *sequence was excluded from clade A, as it was in one of two alternate trees obtained from the AIC analyses. Regardless of its precise affiliation, in an expanded phylogenetic analysis that includes 180 published *gypsy *sequences, *D. virilis *clearly belongs to a well-supported clade that includes all of the *obscura *sequences (see below).

Sequences from *S. elmoi *fall into clade B, in which they form two subclades. Although not monophyletic, the average amount of sequence divergence between the two subclades is just 1.1%, and if the sequences from *S. elmoi *are constrained to be monophyletic the total length of the parsimony tree is unchanged. If the *gypsy *sequences from *S. elmoi *are excluded from the analysis, the topology of the *obsucra *sequences is unchanged from that shown in figure 4. To make the *gypsy *phylogeny conform completely to the species phylogeny (including *S. elmoi *as an outgroup, a monophyletic clade that includes *D. hydei *and *D. virilis*, and *D. melanogaster *as a sister taxon to the *obscura *sequences) increases the parsimony tree length from 1477 to 1542 steps. Thus, there is clear incongruence between the *gypsy *phylogeny and that of the species from which the sequences were isolated.

The envelope sequences from *S. elmoi *differ on average by only 2.1% from those found in the *obscura *species of clade B. As a hallmark of horizontal transfer is the existence of highly similar sequences in the genomes of divergent taxa [15], transfer of *gypsy *from the *obscura *group to the genus *Scaptomyza *seems likely. Because the sampling from *S. elmoi *is quite extensive, an explanation of the retention of an ancestral polymorphism for the phylogenetic incongruence seems unlikely. This conclusion is further supported by comparison to the divergence of a non-mobile nuclear gene, *Adh*. Over exon 2 of this gene, the average divergence between *S. elmoi *and the clade B *Adh *sequences is 20.0%. A horizontal transfer involving the genus *Scaptomyza *and the Palearctic members of the *obscura *species group is of particular interest because of the well-documented horizontal transfer of the P transposable element between these taxa [17]. In that case, the direction of transfer was almost certainly from *Scaptomyza *to *D. bifasciata*. In the case of *gypsy*, the phylogeny indicates a transfer in the opposite direction.

**Figure 5:**
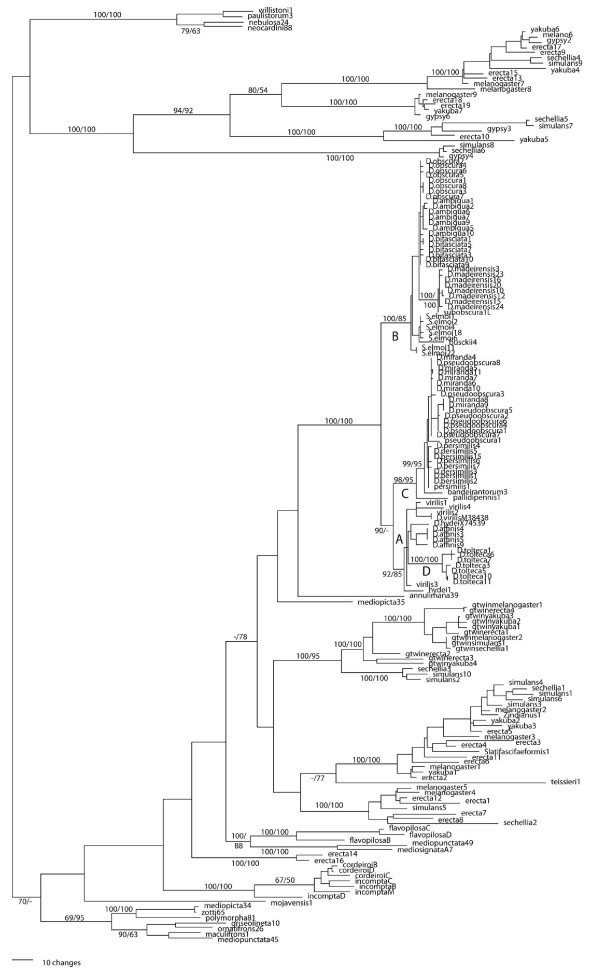
**Neighbor-joining phylogenetic tree of 180 envelope sequences.** Taxon names preceded by a "D." (*obscura *species group) or "S." (*Scaptomyza elmoi*) are from this study. All other sequences are from GenBank, [9] and [26]. Bootstrap values are shown for distance (before slash) and parsimony (after slash) analyses. Values are shown for major clades only. The four clades associated with this study are labeled A, B, C, and D, as in figure 4.

To provide some comparison to other studies of *gypsy *evolution, an extensive phylogenetic analysis was performed with an enlarged dataset of 180 sequences, combining those from this study with those of [9] and [26]. This allows us to assess the coherence of the two clades, A and B, which include postulated horizontal transfer events, and to increase the sample size considerably to examine the alternative hypothesis of ancient lineages. This tree is presented in figure 5. The positions of the *D. hydei*, *D. virilis *and *S. elmoi *sequences remain unchanged from that shown in figure 4, providing additional evidence to support the hypothesis of horizontal transfer.

### Molecular evolutionary analysis of *gypsy *sequences is consistent with activity

To examine the evidence for selection on the *gypsy *sequences, the numbers of synonymous substitutions (*d*S) and nonsynonymous substitutions (*d*N) were calculated for each sequence. The average *d*N/*d*S ratio was then calculated for each clade (see table 2), providing some indication of whether purifying selection is or has been operating on these sequences. For all major clades, the *d*N/*d*S ratio is similar to that obtained for non-mobile nuclear genes [25]. Similar results have been obtained in a number of studies of *gypsy *sequence evolution [6,8-10]. Together, these comparisons suggest that many *gypsy *elements are capable of activity, consistent with the expression data described above. Although the pattern is consistent with activity (and potential infectivity), this conclusion may be complicated by two factors: (i) codon bias, which is lower for the *gypsy *sequences, when compared to *Adh *(data not shown), and (ii) the nature of selection on the *gypsy *sequences which may include functional constraints on the RNA intermediate in addition to selection for a functional envelop protein.

**Table 2:** The ratio of nonsynonymous to synonymous substitutions for the envelope-coding region of the *gypsy *element.

Clade	dN/dS Ratio
A	0.13
B	0.14
C	0.16
D	0.28

Calculations were based on the model of Nei and Gojobori (1986) and include *obscura *sequences only. Clade designations are given in figure 4.

It is noteworthy that clade B includes the *gypsy *sequences that have intact envelope genes (*D. ambigua*, *D. bifasciata*, *D. madeirensis*, *D. obscura*, and *S. elmoi*), whereas clades D and C consist of those with interrupted reading frames. Clade A comprises sequences from the Neartic species *D. affinis*, which also encode full-length envelope proteins and are characterized by very low *d*N/*d*S ratios. It is fascinating that in the two clades with open envelope reading frames and low *d*N/*d*S ratios, horizontal transfer has likely occurred. Even though the *gypsy *sequences examined from clade C are not likely functional, the low *d*N/*d*S ratio suggests that these elements have been active in the recent past. The envelope coding regions of *D. tolteca *(clade D) are characterized by a substantial deletion at the beginning of the envelope gene and relatively higher *d*N/*d*S ratios, suggesting that they have been inactive for considerably longer than the clade C elements. Thus, the phylogenetic and sequence analyses together provide a snapshot of the evolutionary history of *gypsy *in the *obscura *species group that is consistent with their activity and potential infectivity.

Horizontal transfer of *gypsy *has been suggested in other studies: among sequences from the *obscura *group and *D. virilis*/*D. hydei*, which belong to a different subgenus [7], and within the *melanogaster *species group [6]. Two additional studies, which are similar is scope to the one described here, concluded that horizontal transfer of *gypsy *was fairly widespread among species in the genus *Drosophila *and sister genera [9,26]. As these studies included a total of only three sequences from the *obscura *group, they were not able to identify precisely the species involved in the transfer events. In the analysis presented here, the envelope sequence from *D. hydei *shows a strong affiliation to the sequences from *D. affinis*; a less well-supported clade includes the sequences from *D. virilis *as well. As the envelope sequences from *D. hydei*, *D. virilis*, and *D. affinis *differ by an average of only 6.2%, and the species sampling is now quite extensive (see figure 5), horizontal transfer remains the best explanation for the incongruent phylogeny. The hypothesis of horizontal transfer finds additional support in a comparison of the divergence of the envelop region to the non-mobile gene, *Adh*. The average divergence of *Adh *sequences from *D. hydei/D. virilis *and *D. affinis *is 21.4% (clade A).

Using the values for synonymous nucleotide substitutions, [26] provided an estimate of 6.3 million years ago (MYA) for a transfer from the *obscura *group (as represented by *D. persimilis*) to *D. virilis*. When a similar analysis is done with the *D. affinis *sequences (which are not included in [26]), an estimate of only 2.7 MYA is obtained for a transfer from *D. affinis *to *D. hydei*, and 3.1 MYA for a transfer from *D. affinis *to *D. virilis*. In clade B, time estimates calculated from synonymous changes place the transfer from the Paleartic *obscura *to *S. elmoi *at 2.1 MYA. The fact that these transfers occurred relatively recently supports the idea that at least some genomes in the *obscura *group harbor active, infectious *gypsy *elements.

## Conclusion

The genomes of six of the eleven species of analysed encode intact copies of the envelop coding region of gypsy and retain intact envelop splice sites. Their potential for expression was analyzed in vitro and each was found to encode a protein whose size is consistent with activity. Moreover, these elements possess two additional features of active *gypsy *genes, alternative splice sites and the use of alternative start codons. Evolutionary analysis of the envelop sequences shows that interspecies transfer of *gypsy *is associated with those *obscura *elements that can encode a functional envelop protein. Such horizontal transfer is supported by the incongruence of the *gypsy *and species phylogenies and by the low levels of *gypsy *divergence when compared to the divergence of host genes from the same species.

## Methods

### *Drosophila *and *Scaptomyza *stocks

Ten species from the *obscura *group (*D. madeirensis, D. obscura, D. ambigua, D. bifasciata, D. pseudoobscura, D. persimilis, D. miranda, D. affinis, D. algonquin, D. tolteca*), and one species from the *Scaptomyza *genus (*S. elmoi*) were analysed. All species were obtained from the Tucson Stock Center (table 1).

### PCR amplification of the *env *coding region

Genomic DNA from each species was prepared as in [7]. PCR was usually carried out using 100 ng of genomic DNA, 10 pmol of each primer, 10 mM dNTPs and 0.5 U of *Netenzyme *DNA polymerase. The amplifications were performed in a Eppendorf Mastercycler 5333 version 2.01.33-09. The amplified 1443-bp fragment contains a short intergenic stretch, and the entire ORF3 region from the *gypsy *element. The pairs of primers used to amplify the *env *region in the differents species were (see figure 1): *ENV5'mad*: 5'-AGTAGAGTTAGAATAACGTCC-3' and *GYPSY3'*: 5'-TRGCGMGTCAGCATTGTT-3' (where R is G or A) for *D. madeirensis*, *D. ambigua*, *D. bifasciata *and *S. elmoi*; *ENV5'obs*: 5'-ATTAGAGTTAGAACAACGT CT-3' and *GYPSY3' *(see sequence above) for *D. obscura*; *ENV5'pseuaff*: 5'-CTTAGAGTTAGAACACCGTCT-3' and *GYPSY3' *for *D. pseudoobscura, D. persimilis, D. affinis, D. algonquin*; *ENV5'mir*: 5'- CTTAGAGTTAGAACACCAT CT-3' and *GYPSY3' *for *D. miranda*; *ENV5'tol*: 5'- CTTAGAGTTAGAACACCTTCT-3' and *GYPSY3' *for *D. tolteca*.

PCR conditions consisted of a single denaturing step at 94°C for 5 min, followed by 30 cycles at 94°C for 30 s, 55–60°C for 1 min (depending on each primer), and 72°C for 1 min 30 s; a final extension was performed at 72°C for 10 min.

To amplify the 5' region of *gypsy*, which contains the *env *splicing site, different primers, derived from alignments of *gypsy *elements from *D. subobscura, D. virilis *and *D. melanogaster *were designed. The size of the amplified fragment is 500 bp. The sequences of these primers were (see figure 1): *GYPSY5'F*: 5'-GCKWTGATGGCGTATGCATTG-3' (where K is G or T and W is A or T) and *GYPSY5'R: *5'-YTATGCTGCCGAAAGTATGC-3' (where Y is C or T).

### Cloning and DNA sequencing

PCR products were cloned into pCR^®^2.1-TOPO^® ^(Invitrogen). Eight clones from each species were sequenced in an automatic ABI-Prism sequencer. The multiple alignments of *gypsy ORF3 *sequences were performed with the program SEQUENCHER version 4.0.5 (Gene Codes Corporation). The sequencing reactions were carried out with the primers (see figure 1): *ENV*5' and *GYPSY*3' (see sequence above) *GS05*, 5'-TAA TAC TCA CGA TAA CGT TGG-3' for *D. bifasciata*, *D. madeirensis *and *D. ambigua*, *GS04*, 5'-TAA TAC TCA CCA TAA CGT TGG-3' for *D. affinis *and *GS06*, 5'-ATG TCC GAT GAT GTT TAG GAG-3', *GS02*, 5'-CACTTAAATTCAACTTTGGGG-3' and *GS03*, 5'-CCAAAGTTGAATTTAAGTGCC-3' for all species.

All sequences obtained in this study were submitted to the EMBL Nucleotide Sequence Database [EMBL: AM748829 – AM748898].

### Phylogenetic and sequence analysis

Nucleotide sequences were aligned using the default settings of ClustalW http://www.ebi.ac.uk/clustalw/ and were then refined by eye to correct obvious misalignments. The phylogeny of 64 *gypsy *sequences from the *obsura *group and seven sequences from *Scaptomyza elmoi *was examined using parsimony, distance, and maximum likelihood methods as implemented by PAUP* 4.0b10. Four additional *gypsy *sequences, from *D. subobscura *[X72390], *D. hydei *[X74539], *D. virilis *[M38438], and *D. melanogaster *[Z31368] were also included in the analysis. A total of 1392 positions were analysed, with gaps treated as missing data. Identical tree topologies were obtained for all major clades using each method, and adjusting the search parameters for each. The Neighbor-Joining tree shown in figure 4 was constructed using the Kimura two-parameter model for nucleotide substitution. The parsimony analyses, which included 553 informative characters, used random addition of sequences (10 replicates) and TBR branch swapping; both heuristic and branch-and-bound searches yielded identical trees. The maximum-likelihood analyses used empirical base frequencies and a substitution model in which all rates were equal.

Previous studies of *gypsy *phylogeny indicate that the *gypsy *sequence from *D. melanogaster *is an appropriate outgroup for phyolgenetic analysis of sequences from the *obscura *group (e.g., [26]). The use of this taxon as an outgroup was verified in two additional ways. First, it is clearly the most divergent of the *gypsy *sequences, showing an average distance of 36% (Kimura two-parameter method) from the other sequences. Second, midpoint rooting with both neighbor-joining and parsimony establish the root along the *D. melanogaster *branch. Bootstrap values were determined from 1000 replicates for both parsimony and Neighbor-Joining. Character state analyses and parsimony tree-length comparisons were determined with MacClade 4.0.3. The potential effect of recombination on the phylogenetic analyses was assessed with the GARD method [27].

Molecular evolutionary analysis was implemented with MEGA4 [28]. Nucleotide divergences were calculated using the Kimura two-parameter method and the numbers of synonymous substitutions (*d*S) and nonsynonymous substitutions (*d*N) were calculated using the Nei and Gojobori (1986) [29] codon model for the nucleotide sequences that had been aligned based on amino acid position. To facilitate comparisons, the alignments were adjusted by eye to maintain homologous codon positions. Because of some variability in identifying the start site in all sequences, the comparison began at a conserved serine codon, which, in most sequences, is the eighth amino acid of the envelope polypeptide. The divergence of *gypsy *sequences was compared to those of a non-mobile nuclear gene, *Adh*. Sequences for *D. hydei, D. virilis, S. elmoi*, and all available taxa of the *obscura *species group were obtained from GenBank. The comparisons were limited to exon 2, the only sequence available for some species.

### In vitro translation of the Env proteins

To generate PCR products for *in vitro *protein expression, special primers, consisting of two different parts, were designed. The first half adds regulatory elements necessary for expression in a prokaryotic system, such as a T7 promoter, the ribosomal binding site and the T7 terminator. The second half recognizes a specific region of *env*, which corresponds to the sequence used to amplify the *env *region in the different species (see sequences above). This PCR product can be expressed directly in *E. coli *HY using the Rapid Translation System (RTS) from Roche. The *E. coli *Linear Template Generation Set, His-tag and the RTS 100 *E. coli *HY (both purchased from Roche), were used to detect mutations that lead to the termination of mRNA translation and subsequently to protein truncation. The translation products were separated by discontinuous SDS-PAGE through a 12% separating gel with Tris-glycine buffer. The signals were detected by colourimetric system.

### RT-PCR *env *amplification

RNA extractions were carried out using approximately 100 mg of flies. These RNA extractions were carried out with the QuickPrep™ Micro mRNA Purification Kit (Amersham Biosciences), according to the manufacturer's instructions. Afterwards, the mRNA was treated with RQ1 RNase-free DNase (Promega) to remove remainder DNA of the sample. RNA obtained was reverse transcribed in the presence of AMV-RT (Promega) and the *env*-specific primer GS06 (see sequences above).

Reactions were carried out at 37°C for 2 hours followed by 10 minute at 65°C to denature the enzyme. From the first strand reaction, 2 μl was used as a template in PCR using the following primers: GS05, for *D. bifasciata*, *D. madeirensis *and *D. ambigua*, GS04, for *D. affinis *and GS06 (see sequences above) for all species (figure 1).

To analyse the alternative splicing product, the RNA obtained was reverse transcribed and amplified with GS05 and the primer GS06.

## Authors' contributions

JVLL carried out the molecular genetic studies, the obtention of the sequences, participated in the sequence alignment and analysis and drafted the manuscript. JBC carried out the phylogenetic and sequence analysis and drafted the manuscript. IMG participated in the sequence alignment. SS participated in the submission of the sequences to the EMBL Nucleotide Sequence Database and the draft the manuscript. RF participated in the conception and the design of the study. MJMS conceived and designed the study and coordinated and helped to draft the manuscript. All authors read and approved the final manuscript.
